# Diversity in protein domain superfamilies

**DOI:** 10.1016/j.gde.2015.09.005

**Published:** 2015-12

**Authors:** Sayoni Das, Natalie L Dawson, Christine A Orengo

**Affiliations:** Institute of Structural and Molecular Biology, UCL, 627 Darwin Building, Gower Street, WC1E 6BT, UK

## Abstract

Whilst ∼93% of domain superfamilies appear to be relatively structurally and functionally conserved based on the available data from the CATH-Gene3D domain classification resource, the remainder are much more diverse. In this review, we consider how domains in some of the most ubiquitous and promiscuous superfamilies have evolved, in particular the plasticity in their functional sites and surfaces which expands the repertoire of molecules they interact with and actions performed on them. To what extent can we identify a core function for these superfamilies which would allow us to develop a ‘domain grammar of function’ whereby a protein's biological role can be proposed from its constituent domains? Clearly the first step is to understand the extent to which these components vary and how changes in their molecular make-up modifies function.

**Current Opinion in Genetics & Development** 2015, **35**:40–49

This review comes from a themed issue on **Genomes and evolution**

Edited by **Antonis Rokas** and **Pamela S Soltis**

For a complete overview see the Issue and the Editorial

Available online 3rd November 2015

**http://dx.doi.org/10.1016/j.gde.2015.09.005**

0959-437X/© 2015 The Authors. Published by Elsevier Ltd. This is an open access article under the CC BY license (http://creativecommons.org/licenses/by/4.0/).

## Introduction

Families of proteins arise through speciation (orthologous relatives) and through duplication of genes during evolution (paralogous relatives) and it is the paralogues that are most likely to diverge, although not necessarily [1]. By classifying families, superfamilies and collating information on their protein structures, sequences and functions, we can explore how relatives diverge and understand the molecular mechanisms underlying any functional changes [2]. Such insights are essential for inheriting properties between relatives to cope with the huge dearth in experimental annotations. For example, an inspection of the experimental annotations in the UniProtKB/Swiss-Prot sequence database (June 2015) reveals that less than 15% of human proteins have detailed functional characterisation and only 4% have known structures. They are also essential for understanding whether genetic variations are likely to be tolerated and affect function.

**Figure 1:**
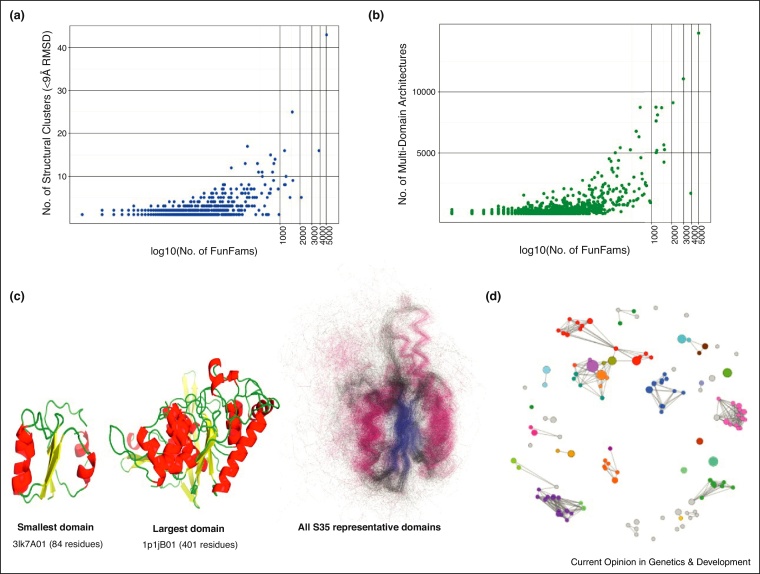
Diversity in protein domain superfamilies. **(a)** Correlation of structural diversity with functional diversity in CATH domain superfamilies. Each point represents a CATH superfamily. Structural diversity is given by the number of distinct Structurally Similar Groups (SSGs) in which relatives superpose with <9 Å RMSD. Functional diversity is given by the number of functional families (FunFams), identified using HMM based strategies [22], and is plotted in the logarithmic (log_10_) scale. **(b)** Correlation of multi-domain architecture (MDA) diversity with functional diversity in CATH domain superfamilies in the logarithmic (log_10_) scale. Each point represents a CATH superfamily. MDA diversity is given by the number of different multi-domain architectures containing one or more superfamily domains. **(c)** Structural diversity in the highly populated ‘NAD(P)-binding Rossmann-like’ superfamily (CATH 3.40.50.720). The figure shows structures of the smallest and largest domain in the superfamily. On the far right is the superposition of all non-redundant superfamily members to highlight the conserved structural core. **(d)** Visualization of functional diversity in the HUP domain superfamily (CATH 3.40.50.620) using Cytoscape [80] networks. The nodes (represented as circles) represent functional families and the edges represent HMM-based family similarities. Each colour denotes a unique Enzyme Commission (EC) number and grey nodes indicate FunFams without any EC number [22].

Many resources now exist for classifying protein families, some of which consider the entire protein (e.g., PANTHER [3], HAMAP [4], TIGRFAMs [5] and SFLD [6]) whilst others classify the domain components (e.g., Pfam [7], SMART [8], PRINTS [9], InterPro [10], CDD [11], CATH [12], SCOP [13] and ECOD [14]) generally considered to be evolutionary independent modules having distinct functional properties [15]. Some resources like PhyloFacts [16] also provide classification of both full-length proteins and domains. At least two thirds of eukaryotic and more than a half of prokaryotic proteins are composed of multiple domains [17] and the most highly populated domain superfamilies are universal to all kingdoms of life or major clades or branches [18]. Therefore, whilst studies have suggested that there may be approximately 100 thousand protein families [16, 19] many proteins can be decomposed into common constituent domains derived from a more limited repertoire of ∼15,000 superfamilies [19]. Within a protein, the different domains tend to have different roles, which when combined make up the general function of that protein. Therefore, by understanding the different functional roles that domains possess we can start to build up a ‘domain grammar of function’ [20]. Interestingly, a few hundred of these domain superfamilies’ dominate nature, accounting for nearly two thirds of all known domains [21]. It is in these superfamilies that we see the most diversity (see Figure 1) and this is largely reflected in their binding properties and/or their ability to metabolise diverse substrates.

In this review we use the CATH-Gene3D domain classification, currently the most comprehensive structure-based superfamily resource, to assess the extent of divergence across protein domain ‘superfamily space’ and review the mechanisms of divergence revealed by detailed studies of specific families undertaken by us and other groups.

## Capturing information on structural and functional diversity within superfamilies

Specialised manually curated structure-based classifications like SFLD [6], TEED [23], CYPED [23], LccED [24] and ESTHER [25] provide valuable insights into the diversity of selected enzyme superfamilies and there have been several elegant studies of large, diverse superfamilies in the Structure Function Linking database (SFLD) resource [26, 27•]. However, relatively few superfamilies have been explored in such detail because of the limited experimental data. Since relatives sharing structural and functional properties experience similar constraints on their sequences to preserve these properties, one way to explore diversity across ‘superfamily space’ is to exploit the much more prolific sequence data that is available [22, 23, 28••].

**Figure 2:**
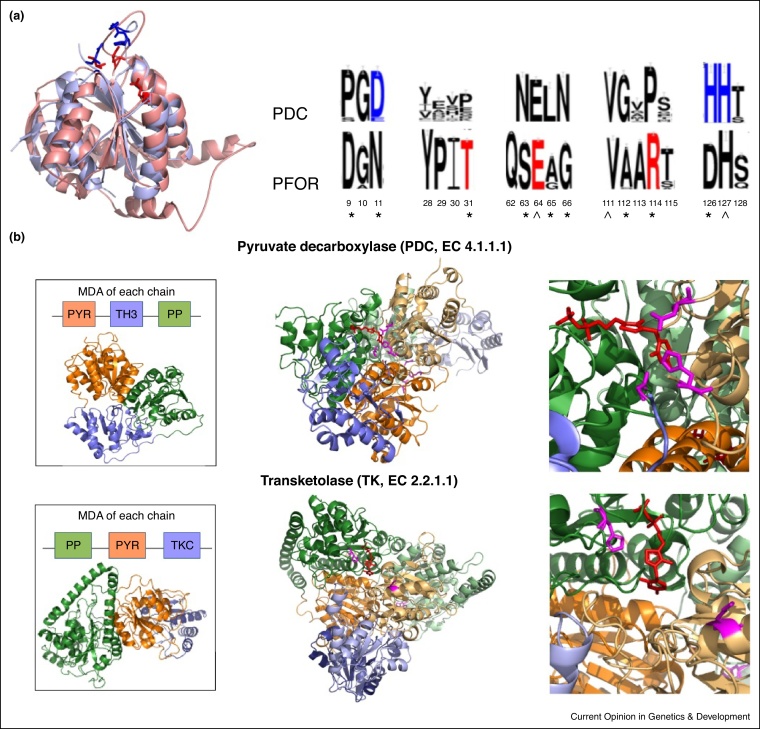
Functional diversity in the Thiamine pyrophosphate (TPP)-dependant enzyme superfamily (CATH 3.40.50.970) due to: **(a)** changes in residues. The superposition of the PYR domains of the Pyruvate decarboxylase (PDC, EC 4.1.1.1) (shown in blue) and Pyruvate:ferrodoxin oxidoreductase (PFOR, EC 1.2.7.1) (shown in red) structures highlights the differences in their catalytic residues (shown as sticks). The specificity-determining positions (SDPs, indicated by an asterisk) around the known catalytic residues are displayed in sequence logos corresponding to the PDC and PFOR functional family in CATH-Gene3D. The catalytic residues are shown in blue for PDC and in red for PFOR and the conserved residues are indicated by a caret (^). The positions are numbered according to the corresponding residue in PDB 1PVD. **(b)** Changes in domain context. Pyruvate decarboxylase (PDC, EC 4.1.1.1) and transketolase (TK, EC 2.2.1.1) in the TPP-dependant superfamily both consist of two chains comprising two TPP domains – PP and PYR (chains are represented by darker and lighter shades of each constituent domain colour). The left hand image shows the difference in multi-domain architectures and 3D arrangements for these two proteins. The middle image shows the different dimeric assemblies that the proteins form. The right image zooms in on the active sites. The TPP molecule is shown in red and the catalytic residues are shown in magenta. Catalytic residues are contributed from the PP domain of one subunit and the PYR of the other subunit. In TK the size of the active site pocket is larger.

By appropriately clustering relatives with similar sequence properties, several resources [6, 16, 19] classify specific ‘functional families’. Approaches range from pairwise comparisons [6] to more sophisticated profile-based analyses [22] that can also be used to detect key residue sites differing between the functional families. Whilst residues important for folding or stability tend to be conserved across the whole superfamily, positions only conserved in certain functional families (specificity determining positions or SDPs) are often under positive selection and associated with distinct functional properties [29, 30] (see Figure 2(a)). SDPs can be associated with a wide variety of protein sites. For example, in addition to mutations in the ligand binding pocket, diversity in the Metabotropic Glutamate Receptors is conferred by SDPs in allosteric sites, the dimerization interface and the hinge region [31^•^]. Similarly the functional specificity of signalling proteins like the Ras superfamily involves mutations in the nucleotide-binding pocket and interfaces co-ordinating the communication between the nucleotide and membrane-binding regions [32].

For exploring superfamily diversity in the CATH-Gene3D resource, we have used an approach that searches for SDPs to distinguish between different functional clusters [22]. This approach sub-classifies the ∼2700 CATH-Gene3D superfamilies into ∼110,000 functional families by optimal partitioning of hierarchical clustering trees for each superfamily, based on identifying characteristic patterns of differentially conserved positions (SDPs) and conserved positions between different functional groups, all of which have at least one relative with an experimental functional annotation in the Gene Ontology (GO) [33]. Whilst validation suggests that these functional groups are reasonably effective in transferring experimental annotations between relatives, there is still considerable room for improvement, as suggested by the results of a recent international large-scale protein function prediction assessment [34]. However, functional family classification does shed light on superfamily diversity, revealing that for only 7% (∼200) of these superfamilies, sequence change is associated with very significant diversity in structure, function and protein context (see Figure 1) while the remaining ∼93% of the superfamilies appear to have structurally and functionally conserved relatives.

## Functional diversity in binding and enzyme superfamilies — ‘molecular tinkering’

Of the 200 most diverse domain superfamilies, each of which have 100 or more functional families and account for ∼50% of all CATH-Gene3D domains, ∼95% of these are superfamilies directly or indirectly associated with enzymatic activity and many of the remainder have relatives with binding activity. Whilst detailed studies of some superfamilies have characterised considerable structural divergence modifying functional site features ([35, 36], see also below), just small changes associated with residue mutations in a binding or active site can alter the shape, physicochemical and electrostatic characteristics significantly, modifying ligand specificities in binding proteins and affecting substrate specificities, chemistries and catalytic efficiencies in enzymes. The Nuclear Receptor superfamily shows amazing diversity in the ligand binding cavity brought about by such mutations, driven by strong divergent selection and adaptive positive selection [37]. Similarly, in the Tubulin superfamily, many of the positively selected sites are found at or adjacent to functionally important sites [38].

In enzymes, considerable sequence divergence can occur in the active sites. In nearly 55% of 101 experimentally well-annotated enzyme superfamilies (accounting for almost 50% of all enzyme sequences in CATH-Gene3D) dramatic changes in catalytic machinery occur [39]. However, in support of previous studies of Babbitt and co-workers [28^••^] which reported that many relatives in SFLD superfamilies share a common mechanistic step, 40% of these superfamilies have one or two catalytic residues common to all functional families. In some cases catalytic residues with similar physicochemical properties are located at similar 3D locations even though they are in different positions in the sequence (see Figure 2(a)). Thus, frequently some aspect of the chemistry is conserved and analyses based on phylogenetic trees derived from structure-based alignments of CATH-Gene3D superfamilies confirm, on a much larger scale than early studies [2], that most superfamily diversity is associated with changes in substrate specificity [40^•^], suggesting that it is hard to change the chemistry presumably because of the complex sequence of mutations needed to create a new arrangement of catalytic residues with the correct spatial relationships.

**Figure 3:**
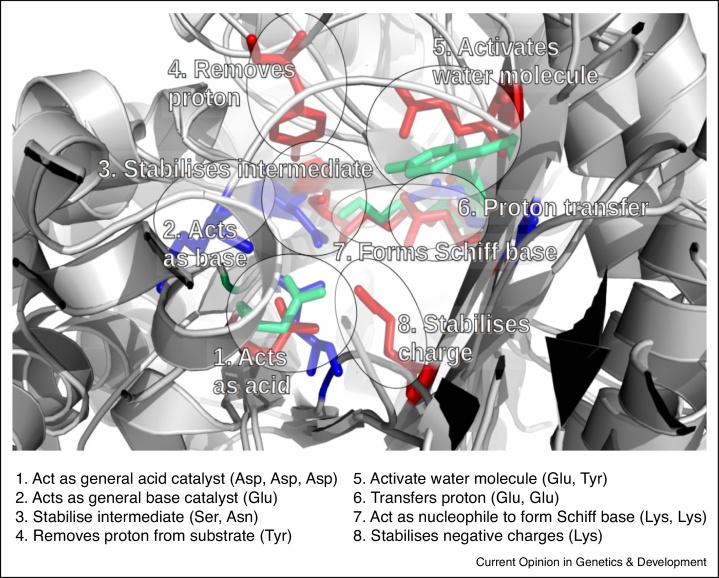
Different catalytic machinery performing same enzymatic chemistry. The three domains shown in this figure use different catalytic machineries to perform the same enzymatic reaction (EC 4.1.2.13). Each domain belongs to a different functional family in the Aldolase Class I superfamily (CATH 3.20.20.70). On the figure different regions in the active site are assigned to clusters 1–8. The catalytic residues in each cluster are reported to have the same functional properties, summarised on the figure. Each colour represents the catalytic residues of a different domain: red is 1aldA00, blue is 1b57A00, and green is 1ok4A00. The remaining portions of the three domains are coloured grey. The same catalytic residue is used by two or more domains in clusters 1, 6, and 7. Different catalytic residues are used in clusters 3 and 5 but still show enough physicochemical similarity to provide the same functionality. The proteins have different catalytic rates which may reflect their different catalytic machineries [51].

However, dramatic changes in chemistry can occur, such as in the Enolase superfamily [41, 42], Aldolase Class I superfamily [43^••^] (see Figure 3) and DRE-TIM metallolyase superfamily [44], and sometimes the same catalytic core is used for very different reactions. For example, many diverse enzymes (peptidases, thioesterases, lipases) in the α/β-hydrolase superfamily use the same catalytic triad (Ser-His-Asp) for different types of bond changes [25]. Diversity can also result from loss or changes to metal ions bound by relatives [45^•^] for example in paraoxonase-1 where an alternative binding mode of the catalytic calcium ion appears to initiate divergence in enzymatic activity [46] and other cases where alterations from the “native” metal of a metalloenzyme have been seen to promote promiscuity [47].

Interestingly, in some enzyme superfamilies, functional families with significantly different catalytic machineries have highly similar functions and substrates, suggesting either convergence within the superfamily or evolutionary drift from a common functional ancestor along different routes, that is, perhaps a trajectory to a less efficient enzyme with subsequent mutations restoring the activity or even resulting in a more efficient form of the enzyme. It is difficult to distinguish these cases without robust phylogenetic analyses. Such studies on Rubisco, an abundant protein important for carbon fixation, show that a more efficient form of Rubisco has emerged by convergent evolution more than 62 times in harsh environments, and structure-based analyses reveal mutations in the active site loop and secondary shell, where they possibly influence rearrangements of the active site; also at interfaces in the oligomer suggesting a role in allostery [48^•^].

Enzyme superfamilies showing the greatest versatility in CATH-Gene3D, frequently adopt alpha/beta structures, two thirds having TIM or Rossmann folds. As Tawfik and his colleagues have reported in a recent publication, these structures tend to have regular, well-packed structural cores and the catalytic residues mainly locate to loops largely detached from these cores and therefore perhaps better able to tolerate the destabilising effects of mutations [49•, 50••].

Diversity in protein superfamilies can also arise from mutations in protein interfaces. Furthermore, relatives can exploit completely different surfaces in their protein interactions. Large-scale studies comparing CATH-Gene3D functional families showed that in 645 highly versatile superfamilies, cumulative binding sites from diverse relatives covered most of the protein surface and were associated with a wide range of protein partners [52^•^]. However, sometimes the same interface is exploited but by different partners. In the two Dinucleotide Binding Domains Flavoproteins (tDBDF) superfamily, the diversity of reactions carried out by relatives is achieved by different protein partners acting as electron acceptors and interacting with the same face of the tDBDF domain [53]. Paralogous relatives are more likely to bind different protein partners [54] and this is a significant effect in the beta-propellor superfamilies, whose structures contain repeating WD40 sub-domains, and in which human paralogues have multiple distinct surfaces interacting with a very wide variety of proteins, peptides or nucleic acids [55].

## Structural mechanisms of superfamily divergence

Although only 10% of the CATH-Gene3D functional families have structural representatives, this data can help identify superfamilies capable of great structural plasticity where relatives display considerable diversity due to extensive residue insertions and repetitions or inserted structural motifs [56, 57]. For ∼160 CATH superfamilies, accounting for half of all known domains in CATH-Gene3D, at least a two-fold variation in the size is observed between the most diverse domains [58]. However, analyses of selected superfamilies [35, 59] and more recent large-scale studies have shown that the structural core (generally 40–50% of the domain) is highly conserved even for relatives separated by billions of years [57] (see Figure 1). Long residue inserts in diverse relatives generally adopt secondary structure features that form structural decorations to this core and can be associated with modified functions, for example, by altering active site geometry and thereby changing substrate specificity (see Figure 2(a)), or altering surface features and thereby changing protein interaction partners [52^•^]. In the Thiamine pyrophosphate (TPP)-dependant superfamily, different functional families have varying inserts forming small additional secondary structure features that reshape the active site for different substrates (see Figure 2(b)). In the HUP domain superfamily, also, quite extensive structural embellishments extend the active site [35]. Insertions of motifs or sub-domains can also result from gene fusions, for example, in the Haloalkanoic Acid Dehalogenase (HAD) superfamily where they provide diverse specificity determinants for a broad range of substrates [60^••^].

Dramatic structural rearrangements can also arise from variations in repeating units. In the Vicinal Oxygen Chelate (VOC) superfamily, members share a common βαβββ subdomain that is organized into different topological (or domain-swapped) combinations in different relatives that maximizes the catalytic versatility of the metal center [61]. These and other structural changes such as circular permutations and rearrangements in β-sheet topologies can sometimes transform the fold [62] as well as modifying the function [50^••^].

Diversity can also emerge from changes in less structured regions, for example, repeats giving rise to low-complexity regions (LCRs), such as polyalanine or polyglutamine runs. These often evolve rapidly and can have a major influence on the transcriptional activity of the protein [63]. Similarly, variations in (Gly)n -X repeats in glycine rich domains have been observed to alter the expression pattern, modulation and sub-cellular localization of relatives in some plant families [64].

## Superfamily diversity arising from different multi-domain contexts

Gene fusions are another evolutionary mechanism conferring diversity as they can significantly alter the context of a domain (i.e., by changing the multi-domain architecture (MDA) of the protein), thereby modifying its molecular function and biological role. Domains have been frequently duplicated and shuffled within genomes, during evolution, with fusions being more frequent and generally occurring at N or C termini [65]. For 92% of the 200 most diverse superfamilies in CATH-Gene3D superfamilies, that is, those having the highest number of functional families, relatives occur in more than 100 different multi-domain contexts [21] (Figure 1(b)). Changes in domain partners may not necessarily alter the function of the domain but change the context in which it operates, for example, locating it in different protein complexes and/or pathways. For example, early studies demonstrated the recruitment of domain relatives to different metabolic pathways for the chemistry they bring [66].

However, changes in domain partners can also alter specificity. For example, in the highly diverse Thiamine pyrophosphate (TPP)-dependant enzyme superfamily changes in domain partnership alter the size and physicochemical properties of the active site pocket (see Figure 2(b)), enabling a huge range of substrates, products and stereo-selectivity [67]. Different oligomerisation states also effectively change the domain context. Again, in the TPP superfamily, various oligomerisation states have evolved in different species. Whilst some may be associated with enhanced stability, others clearly influence active site characteristics by changing the positioning of the domains providing catalytic residues (see Figure 2(b)).

## Diversity in superfamilies due to promiscuity

Diversity within a superfamily can also be the result of individual relatives having multiple functions. For example, relatives can have multiple catalytic activities not necessarily of equal efficiency, as in promiscuous enzymes; or moonlighting functions whereby proteins perform completely different functions to their native activity sometimes involving different sites [68, 69]. Promiscuity can be the starting point for the evolution of a new function [49•, 50••]. Under natural selection, promiscuous enzymes can give rise to specialist enzymes by a variety of different mechanisms - protein dynamics (e.g., changes in conformational dynamics have converted a promiscuous generalist beta-lactamase to a penicillin-specific beta-lactamase, without significant changes in the structure of the active site [70]), domain insertions (e.g., HAD superfamily [36, 60••]), rearrangements in the catalytic metal ions [71] and binding of alternative cofactors [72].

An increasing number of proteins are now known to moonlight and these activities can be induced by oligomerisation, cellular localization, differential expression and substrate concentration. For example, Albaflavenone monooxygenase in the Cytochrome P450 superfamily, also functions as a Terpene synthase, an activity not observed in any other superfamily member. The catalytic machineries for the two enzymatic reactions are located in distinct pockets on the domain and the reactions are carried out at different pHs [73].

## Conclusions

**Figure 4:**
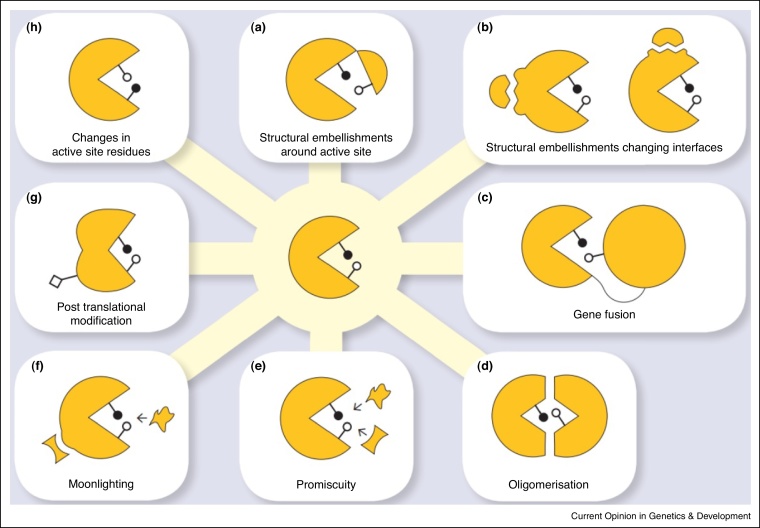
Functional diversity of proteins can arise due to one or more of the following mechanisms: **(a)** Structural embellishments around active site, **(b)** Structural embellishments changing interfaces, **(c)** Gene fusion, **(d)** Oligomerisation, **(e)** Promiscuity, **(f)** Moonlighting, **(g)** Post-translational modification and **(h)** Changes in active site residue. Note that for the mechanism panels **(a)**, **(c)** and **(d)**, one of the enzyme active site residue is contributed by its domain partner.

In most large diverse superfamilies, functional diversity results from a combination of different molecular mechanisms (Figure 4). For example, in the PD-(D/E)XK Phosphodiesterase superfamily there are structural embellishments to the core, domain swapping events, active site residue variations and changes in MDA [74]. Similarly, in the Ribonuclease H-like (RNHL) superfamily [75], and many other families discussed above.

Experimental data on functional diversity grows slowly as detailed studies are time-consuming and expensive, however, classifying the millions of sequences accumulating in public repositories like UniProt into putative functional families can reveal subtle changes in conservation patterns that suggest shifts in binding specificities or catalytic machineries. These data can guide experiments to focus on unusual relatives and more comprehensively landscape the functional repertoires of the most versatile superfamilies. For example, sequence similarity networks based on protein families can help in providing a comprehensive summary of sequence, structure and function relationships in a functionally diverse superfamily. Recent studies [27•, 60••, 76] of such networks derived from curated family classification for three functionally diverse superfamilies in SFLD have been used to aid in target selection for interesting targets for experimental characterisation. The availability of automated functional classifications of superfamilies will ultimately guide experimental validation using high-throughput approaches and aid in improving the functional annotation of genomes. This will be especially important for large diverse superfamilies.

Only ∼63% of the 25 million domain sequences in CATH-Gene3D can be assigned to an experimentally annotated functional family and less than 10% of these families have a known structure, so there may be much more diversity to discover. Certainly analyses of microbial communities hint at exciting novel chemistries [77, 78]. Although the lack of data hinders our understanding, most studies of enzyme superfamilies, even those that are mechanistically very diverse, suggest that chemistry is usually preserved or there is conservation of a specific partial reaction among all relatives and that it is substrate specificity that is much more likely to change [28^••^]. Furthermore, the relative success of domain-based strategies for protein function prediction [22, 79] suggests that a general functional role is conserved across most domain superfamilies and that diversity largely results from exploitation of that role on multiple ligands or substrates, and in multiple contexts. In other words, the structural diversity observed in promiscuous superfamilies is more frequently associated with changes that reflect different domain contexts or changes in substrate specificity rather than dramatic changes in the functional role. This suggests that for many domain superfamilies’ a domain grammar of function can be applied.

## References and recommended reading

Papers of particular interest, published within the period of review, have been highlighted as:

• of special interest
•• of outstanding interest
